# How Does Self-Declared Chronic Pain Compare to Other Definitions? A Prospective Multicenter Study

**DOI:** 10.1155/prm/5556400

**Published:** 2025-06-19

**Authors:** Raoul Daoust, Jean Paquet, Jeffrey J. Perry, Justin W. Yan, David Williamson, Véronique Castonguay, Gilles Lavigne, Dominique Rouleau, Justine Lessard, Alexis Cournoyer, OPUM research group

**Affiliations:** ^1^Department of Emergency Medicine, Research Centre, Hôpital Sacré-Coeur, Montréal, Québec, Canada; ^2^Département Médecine Familliale et Médecine d'Urgence, Faculté de Médecine, Université de Montréal, Montréal, Québec, Canada; ^3^Department of Emergency Medicine, Acute Care Research Program, Ottawa Hospital Research Institute, University of Ottawa, Ottawa, Ontario, Canada; ^4^Department of Medicine, Division of Emergency Medicine, London Health Sciences Centre, Western University, London, Ontario, Canada; ^5^Centre de Recherche de l'Hôpital du Sacré-Coeur de Montréal, Montréal, Québec, Canada; ^6^Faculté de Pharmacie, Université de Montréal, Montréal, Québec, Canada; ^7^Faculty of Dental Medicine and Medicine, Université de Montréal, Montréal, Québec, Canada; ^8^Orthopedic Department, Hôpital Sacré-Coeur, Montréal, Québec, Canada; ^9^Université de Montréal, Montréal, Québec, Canada

**Keywords:** chronic pain, emergency department, self-declared, validity

## Abstract

**Background:** Self-declared chronic pain has not been compared to existing definitions. Our objective was to evaluate the agreement between self-declared chronic pain and different chronic pain definitions, three months after an emergency department (ED) visit.

**Methods:** In this planned substudy of a prospective multicenter cohort study, we included consecutive patients aged ≥ 18 years with an acute pain condition discharged from the ED with an opioid prescription. Three months after their ED visit, participants were asked about their pain intensity, pain frequency, pain disability, and self-declared chronic pain. Agreement between self-declared chronic pain and five other definitions were calculated with kappas.

**Results:** A total of 1411 participants were included; mean age was 52 (±16) years, and 53% were female. Prevalence of self-declared chronic pain was 23.0% and varied from 16.9% to 45.3% for other definitions. Agreement of self-declared chronic pain was moderate (0.57–0.60) with most definitions but lower with the pain intensity ≥ 1 definition (0.47). The proportion of chronic pain participants using opioids ( ⁓20%) or other analgesics (⁓80%) was similar with all definitions except for the pain intensity ≥ 1 definition which was associated with a lower proportion of analgesic use (11%, 64%).

**Conclusion:** In summary, self-declared chronic pain displayed moderate agreement with other chronic pain definitions and similar analgesic consumption but lower with the pain intensity ≥ 1 definition. Nonetheless, chronic pain prevalence varied greatly depending on how it was defined. Self-declared chronic pain might be a more patient-centered outcome and could be easily applied to standardize chronic pain definition.

**Trial Registration:** ClinicalTrials.gov identifier: NCT03953534

## 1. Introduction

Chronic pain is a leading cause of disability, affecting between 20% and 50% of the general population [1, 2], and is one of the most common reasons for seeking healthcare [3]. Chronic pain can be seen as an umbrella term referring to a wide range of painful conditions, such as fibromyalgia, migraine, or long-standing painful state of unclear etiology [4]. Moreover, inadequate acute pain management can also lead to the development of chronic pain [5, 6]. Several emergency department (ED) studies have shown that acute pain intensity after ED discharge at specific time points, and its evolution is associated with the development of chronic pain at 3, 6, or 12 months [7–10].

The International Association for the Study of Pain (IASP) and the International Classification of Diseases, 11th revision, define chronic pain as “pain has persisted for more than 3 months and is associated with significant emotional distress and/or functional disability, and the pain is not better accounted for by another condition” [3, 11]. However, except for the duration of three months, no other threshold is determined by the IASP. This definition differentiates chronic pain (as persisting beyond normal tissue healing time) from acute pain [12]. A meta-analysis of 86 studies on the prevalence of chronic pain and its definitions reported large variations in prevalence, from 8.7% to 64.4% [13]. The duration required to define chronic pain varies from three to six months, with three months being the most frequently used [13]. Despite this, only two of 86 studies used the same operational definition to assess chronic pain incidence or prevalence, leading the authors to conclude that variations in the estimates are largely a direct result of definition inconsistencies [13].

Generally, chronic pain has been identified mostly by its duration from onset. However, studies have proposed other operational definitions. For example, pain intensity threshold [7, 14, 15], pain frequency [1, 16–19], pain-related distress [20–23], and functional disability [24, 25] are often used to operationalize chronic pain [13]. These definitions could influence the association with resource use. For instance, it would be expected that a chronic pain definition based on pain intensity would be associated with pain medication use. However, we could not find this information in the literature. Furthermore, we were unable to identify a study that compared self-declared chronic pain to other definitions.

Considering that there is no clear consensus on the operational definition of chronic pain in the literature and that most definitions rely on self-reported pain intensity, distress, or functional disability, we want to investigate if patients could determine with a simple question, without specific knowledge or explanation, if they suffered from chronic pain. This would be an easy, quick, and patient-centered method to identify these patients and could be associated with better patient-centered chronic pain management [26, 27]. We hypothesized that prevalence would vary greatly between definitions and that self-declared chronic pain would provide similar prevalence and analgesic consumption to definitions associated with at least moderate pain intensity or disability. The objectives of this study are to evaluate the agreement between self-declared chronic pain and different chronic pain operational definitions and the impact on prevalence and analgesic use after a visit to the ED for acute pain.

## 2. Methods

### 2.1. Patient and Public Involvement

A patient partner was involved in the study's design and the conduct of the study and took part in the development of interviews.

### 2.2. Study Design and Setting

This was a planned substudy of a Canadian multicenter prospective cohort study conducted in six tertiary care Level 1 trauma center academic hospitals' ED and in one community hospital's ED: four from the province of Quebec and three from the province of Ontario, Canada. The main study was conducted to determine the optimal quantity of opioids to prescribe to ED discharge patients with acute pain [28]. The annual census across hospitals varies from 43,000 for the community hospital to 150,000 ED visits for the largest tertiary care hospital. Approval was obtained from each local institutional ethics review board.

### 2.3. Participants

Consecutive patients aged 18 years and older, treated in the ED from May 2019 to January 2023 were identified by ED clinicians 24/7, and then recruited by research assistants. Recruitment was halted from March 2020 through October 2020 due to the COVID-19 pandemic. We included patients with an acute pain condition present for less than 2 weeks (usual acute pain definition) and discharged from the ED with an opioid prescription. We excluded patients who did not speak French or English, who were unable to fill out a diary or unavailable for follow-up, were using opioid medication prior to the ED visit, or were suffering from cancer or chronic pain requiring any form of treatment. Finally, for this analysis, we excluded patients with renal colic because this condition rarely evolves to chronic pain [29].

### 2.4. Procedures

ED clinicians indicated the final diagnosis category (renal colic, abdominal pain, fracture, back pain, neck pain, other musculoskeletal, or other) and the prescribed/recommended pain medications. To be broad, we included the “other diagnoses” category for patients with all uncategorized pain conditions (e.g., abscess, burn, tooth pain, head trauma, diverticulitis, chest pain, gout, and shingles). Research assistants verified exclusion criteria and subsequently obtained informed consent from the patient. If no research assistant was available (e.g., nighttime), patients were contacted later by phone to obtain informed consent. Age, sex, pain intensity at triage, opioid use during ED stay, and length of ED stay were extracted from each hospital's computerized medical system. On the first day after ED discharge, participants had to respond to the initial questionnaire which included baseline characteristics. Study participants also self-identified their ethnicity from seven categories based on the 2019 Version of the Canadian Institute for Health Information [30].

Three months after ED discharge, participants were contacted by phone and questioned about their pain and how it affected their normal activities. We first asked the participants the following “yes or no” question: “According to you, would you say that you currently suffer from chronic pain?” Participants who answered “yes” were then asked the follow-up questions. “Is your chronic pain related to your ED visit 3 months ago?” (yes, no). Then, patients were asked about their pain intensity and frequency: “In the past week, what was your average pain on a scale of 0–10: 0 being no pain at all and 10 being the worst pain imaginable?” Participants with a score of at least 1 were then asked: “Is the pain experienced during the past week related to the pain you had when you visited the ED 3 months ago?” (yes, no). “In the past week, how often have you experienced pain related to your ED visit 3 months ago?” (every day, most days, occasionally, and rarely). Participants were also asked if they had consumed opioids, nonsteroidal anti-inflammatory drugs (NSAIDs), or acetaminophen in the past week related to their initial painful condition.

Finally, patients were asked to complete the Pain Disability Index (PDI) [31], which consists of seven categories that are measured on a scale from 0 (no disability) to 10 (all the individual's normal activities have been totally disrupted, or impossible to do, because of pain). Family and household responsibilities, recreation, social activities, occupation, sexual activities, independence, and life support activities make up the seven areas of the PDI. The final score is the sum of all the individual's domain scores: range 0 (no disability) to 70 (extreme disability). Study data were collected and managed using Research Electronic Data Capture (REDCap), a secure, web-based application tool hosted at Hôpital Sacré-Coeur de Montreal [32].

### 2.5. Outcomes

The main outcome of this study was the presence of chronic pain at 3 months after ED discharge. We used six chronic pain operational definitions: (1) Self-declared chronic pain when patients answered “yes,” without specific knowledge or explanation, to the question “According to you, would you say that you currently suffer from chronic pain?” (2) Since there is no consensus on the required threshold for pain intensity to define chronic pain, and to be inclusive, we created an operational definition with a pain intensity of ≥ 1 on a 0–10 scale [25, 33–39]. (3) Pain intensity of ≥ 4 on a 0–10 scale without considering the frequency of pain [7, 14, 15]. (4) Pain intensity of ≥ 4 on a 0–10 scale with a pain frequency of every or most days [1, 16–19]. (5) PDI of moderate or higher disability. Chronic pain can limit daily life activities [24, 25], referred to in some studies as “high impact chronic pain” [40]. To establish our disability threshold, we transformed the PDI final score from a 0–70 scale to a percentage by computing: PDI∗ 100/70. Since the PDI is highly correlated with the Oswestry Disability Questionnaire (ODQ) (*r* = 0.83) [41], we used the same ODQ disability threshold: 0%–20% (minimal disability), 21%–40% (moderate disability), 41%–60% (severe disability), 61%–80% (infirm), and 81%–100% (bedridden). (6) Finally, we combined the following definitions to arrive at the following: pain intensity of ≥ 4 on a 0–10 scale with a pain frequency of every or most days, and moderate or higher disability on the PDI. In all definitions, pain at 3 months had to be related to the initial acute pain. We also assessed at 3 months the impact of these chronic pain definitions on prevalence and analgesic consumption (opioids, NSAIDs, and acetaminophen) in the past week related to the initial acute pain visit.

### 2.6. Statistical Analysis

Descriptive statistics were used to compare baseline characteristics between included and excluded participants using mean with SD (or median ± IQR for non-normal distributed variables) for continuous variables and proportions for categorical variables. A maximum of two missing items was accepted for the PDI. When one or two items of the PDI were missing, the mean score of the participant's other items was calculated. The final score was calculated by replacing the missing item's score by this calculated mean. Prevalence estimates of chronic pain according to different criteria were reported as percentage ± 95% confidence intervals (CIs). Degree of agreement between self-declared chronic pain and the five different chronic pain operational definitions were calculated with kappas ± 95% CI. Cohen suggested the kappa result be interpreted as follows: values ≤ 0 as indicating no agreement and 0.01–0.20 as none to slight, 0.21–0.40 as fair, 0.41–0.60 as moderate, 0.61–0.80 as substantial, and 0.81–1.00 as almost perfect agreement [42]. Comparisons of baseline characteristics for participants agreeing and those disagreeing between self-declared chronic pain and the operational definitions were performed using the chi-square or independent *t* tests. We reported the percentage of participants for each cross-tabulation of self-declared and the different operational definitions of chronic pain. We also presented and compared the percentage of opioid and NSAID/acetaminophen use for each chronic pain definition using the chi-square test. Alpha level was set at 0.05, and all statistics were performed using SPSS Version 23 (IBM, Somers, NY).

In a test for agreement between two chronic pain definitions using the kappa statistic, a sample size of 834 subjects achieves 90% power to detect a difference of 0.10 between H0 and H1 when there are two categories with frequencies equal to 0.25 and 0.75 with a significance level of 0.05 (PASS Version 11.0, NCSS, LLC. Kaysville, Utah). Since we used a large prospective database (OPUM study) to perform this study, we opted to use all available patients that were included in the 3-month follow-up.

## 3. Results

### 3.1. Sample Characteristics

**Figure 1:**
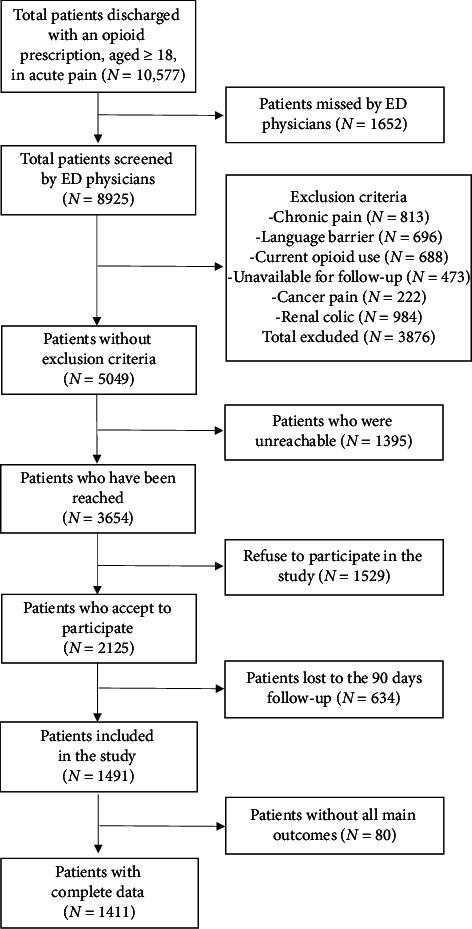
Flowchart of the study.

During the recruitment period, 10,577 patients met our inclusion criteria. Of these, 13% were missed by ED clinicians, 39% were excluded, 13% were unreachable (research assistant unavailable at patient presentation and unreachable after five phone calls for inclusion), 14% declined to participate, 30% of participants were lost to follow-up at three months, and 4% had missing main outcome, leaving us with 1411 participants (Figure 1). Participants and patients lost to follow-up were similar on baseline characteristics, except that participants were more often from White ethnicity and had more college/university degree compared to lost to follow-up patients (Table 1). More details on the cohort baseline characteristics are reported in a previous publication [28]. Most participants were recruited from the Province of Québec's tertiary care sites (78%), 14% from a community center, and 7% from Ontario's tertiary care sites.

Participants' mean age was 52 (±16) years, 53% were female, and mean pain intensity at triage was 7.0 (SD = 2.2). The most frequent pain conditions encountered were fractures, other musculoskeletal pain (contusion, bursitis, strain, muscle/tendon tear, sprain, dislocations, or tendinitis), and back pain. More than half of the participants were treated with opioids during their initial ED visit, and the median ED stay duration was shorter than 6 hours (Table 1). There was no significant difference in the proportion of participants who self-declared suffering from chronic pain between French-speaking participants (22.1%) and English-speaking ones (24.3%; *p*=0.32).

### 3.2. Agreement Between Self-Declared Chronic Pain and the Operational Definitions

The degree of agreement between self-declared chronic pain and chronic pain determination according to different operational definitions are reported in Table 2. There is moderate agreement between self-declared chronic pain and the different operational definitions; the lowest agreement (0.47) was with the pain intensity ≥ 1 operational definition. We examined the baseline characteristics for participants with and without agreement between self-declared chronic pain and the other chronic pain operational definitions (Supporting Tables 1–4). There were some significant age differences between participants with and without agreement, but these differences were not clinically significant. However, participants with fractures or back pain had significantly less agreement than neck pain, other musculoskeletal pain, abdominal pain, or other pain conditions (*p*=0.001).

### 3.3. Chronic Pain Prevalence

The chronic pain prevalence at 3 months after the index ED visit varied from 17% to 45% according to the different definitions (Table 3). As expected, the less strict pain intensity ≥ 1 operational definition provided the highest prevalence, which decreased with the pain intensity ≥ 4 criteria and the addition of other criteria (frequency and disability). The self-declared chronic pain prevalence (23%) was similar to the chronic pain operational definition of pain intensity ≥ 4.

### 3.4. Type of Agreement Between Self-Declared Chronic Pain and the Operational Definitions

More participants identified as suffering from chronic pain using the pain intensity ≥ 1 operational definition did not self-declare as having chronic pain compared to other definitions (24% vs. 4%–10%). Participants that did not meet the pain intensity ≥ 1 operational definition for chronic pain showed low self-declared chronic pain (2%), whereas patients not meeting other chronic pain definitions seemed to show more self-declared chronic pain (6%–10%). On the other hand, the percentage of patients who meet the criteria for both self-declared chronic pain and other definitions was similar (13%–16%), except for the pain intensity ≥ 1 operational definition (22%) (Table 4).

### 3.5. Opioid and NSAIDs/Acetaminophen Use According to Different Chronic Pain Definitions

For participants meeting the pain intensity ≥ 1 operational definition, compared to other definitions, the proportions of opioid and NSAIDs/acetaminophen use related to their initial painful condition were lower, 11% and 64%, respectively (*p* < 0.001). As the chronic pain definition criteria become more stringent for other definitions, the opioid and NSAIDs/acetaminophen use related to their initial painful condition tended to increase slightly from 17% to 21% and 75% to 84%, respectively (Table 5).

## 4. Discussion

In this multicenter prospective study, self-declared chronic pain showed moderate agreement with different operational definitions of chronic pain three months after an ED visit for acute pain. However, the agreement was lower with the pain intensity ≥ 1 operational definition, and disagreement seems mostly driven by fracture and back pain. Less than 10% of participants do not self-declare chronic pain if established as suffering from chronic pain according to most definitions; only the pain intensity ≥ 1 operational definition demonstrated a higher percentage (24%). Furthermore, self-declared chronic pain is associated with similar levels of opioids and/or other analgesic usage than other definitions.

We also showed that the use of different operational definitions of chronic pain affects its prevalence, with almost half of patients meeting the pain intensity ≥ 1 operational definition for chronic pain compared to about a quarter of patients with other definitions. A 2017 review and meta-analysis on the definition of chronic found similar variation in prevalence (8.7%–64.4%), and apart from the 3-month duration of the pain, there was almost nothing in common in the definition of chronic pain across studies [13]. The higher prevalence of the pain intensity ≥ 1 operational definition's in our study may be explained by the low pain intensity threshold criterion (≥ 1 on a 0–10 scale). We expected a lower prevalence of chronic pain with the PDI definition since it implied a criterion of moderate or higher pain-related disability. Since the median pain intensity was low in this group (3 on a 0–10 scale; data not shown), physical limitations associated with fractures and musculoskeletal trauma but unrelated to pain could explain it.

In our study, except for the pain intensity ≥ 1 operational definition, approximately 80% of participants identified as suffering from chronic pain consumed an analgesic and close to 20% still consumed opioids. It has been reported that about 7% of all opioid naïve patients who received an opioid prescription continue using opioids three to 12 months after ED discharge [43]. Also, opioid availability in a given household is associated with increased odds of opioid overdoses in the same household in other persons than the patient who received the prescription [44]. Surprisingly, studies on risk factors of long-term use have not identified chronic pain has a risk factor [45–47]. This could be explained by the retrospective study design of most studies and the fact that chronic pain is underreported in these databases. Our findings suggest that chronic pain could have an impact on the opioid crisis, and the first step in pursuing this research further should be to standardize the chronic pain definition.

There is no consensus on the best operational chronic pain definition. As resources for chronic pain are often limited and access to multidisciplinary chronic pain clinics variable, it would be logical to promote the use of a definition associated with significant pain intensity, medication use, or disability [48, 49]. Self-declared chronic pain seems to provide a similar prevalence of chronic pain and opioids or NSAIDs/acetaminophen use as other chronic pain definitions except for the pain intensity ≥ 1 criteria, which is associated with a higher prevalence and lower analgesic consumption. It is surprising that 10% of patients with the moderate or higher disability criteria did not self-declare chronic pain. Since most pain conditions in our study were related to musculoskeletal injuries including fractures, patients may accept physical limitations and pain as the natural progression of their injury and not consider this to be chronic pain. It has been reported that fracture patients create a new way of living, adapting to their limitations, and that they prioritize regaining their independence and sleep quality [50–52]. Self-declared chronic pain could be a simple alternative to complex operational definitions; these patients could also be more receptive to chronic pain treatment approaches and diligent with the partnership associated with the quality of rehabilitation [27, 53, 54].

### 4.1. Limitations

This study has limitations. Included participants compared to lost to follow-up patients were more often from White ethnicity and had more college/university degree. Most participants were from the Province of Québec, which could affect the main outcome because of different medical practices. Since the number of participants was low for neck pain, caution must be taken for this specific pain condition. Furthermore, all patients in this cohort were prescribed opioids after their ED visit, so other populations who were not prescribed opioids or had less intense acute pain could generate different results. Also, opioid hyperalgesia could in rare instances have a significant impact on pain intensity, and this could produce slightly higher chronic pain prevalence for every definition [55, 56]. Moreover, all self-reported definitions of chronic pain in this study are at the risk of patients exaggerating their symptom due to secondary gains. Nevertheless, our goal was to compare the prevalence and analgesic use of different operational definitions in our opioid-exposed population, not to determine the prevalence for the general population. Our cohort was also well educated; results could be different with less-educated patients. However, we did not observe an association between the education level and agreement level between self-declared chronic pain and other more operationalized chronic pain definitions (supporting tables). We did not have information on psychological distress so we could not compare definition using this information. We only studied analgesic use associated with different chronic pain definitions, and other important outcomes such as quality of life or utilization of health resources could provide different results and should be studied in future research.

## 5. Conclusions

In summary, self-declared chronic pain displayed moderate agreement with other chronic pain definitions and similar analgesic consumption, except for the pain intensity ≥ 1 operational definition which was associated with a lower agreement and a lower analgesic consumption. Nonetheless, the prevalence of chronic pain varied greatly depending on how it was defined. Self-declared chronic pain might be a more patient-centered outcome and could be easily applied to standardize the definition of chronic pain.

## Figures and Tables

**Table 1:** Baseline characteristics of included and lost to the 3-month follow-up patients.

Baseline characteristics	Lost to follow-up (*N* = 714)	Included participants (*N* = 1411)
Mean (±SD) age	48.6 (16.7)	51.9 (15.7)
Female (%)	52.9	52.9
Mean (±SD) pain intensity (0–10 scale) at triage	7.0 (2.2)	7.0 (2.2)
Ethnicity group (%)		
White	69.9	80.2
Other	30.1	19.8
Education (%)		
Primary/high school	42.2	34.6
College/university	57.8	65.4
Type of pain conditions (%)		
Fracture	24.9	29.8
Back pain	24.5	24.5
Neck pain	3.4	3.5
Musculoskeletal	23.1	22.7
Abdominal pain	8.7	9.4
Other	15.4	14.6
Treated with opioid within the ED stay (%)	60.9	55.1
Median (Q1–Q3) ED stay (hours)	5.7 (3.7–8.6)	5.5 (3.6–8.4)

*Note:* Q1–Q3: first and third quartile.

Abbreviations: ED = emergency department, SD = standard deviation.

**Table 2:** Degree of agreement between self-reported chronic pain related to the initial ED pain and different chronic pain operational definitions.

Chronic pain operational definition	Kappa	95% CI
Patient with pain intensity ≥ 1^∗^	0.47	0.43–0.51
Patient with pain intensity ≥ 4^∗^	0.60	0.55–0.65
Patient with pain intensity ≥ 4^∗^, every or most days	0.59	0.54–0.64
Patient with moderate or higher disability level (PDI)	0.57	0.52–0.62
Patient with pain intensity ≥ 4^∗^, every or most days with moderate or higher disability level (PDI)	0.57	0.52–0.62

*Note:* (*N* = 1411).

Abbreviations: CI = confidence interval, ED = emergency department, PDI = Pain Disability Index.

^∗^Pain intensity on a 0–10 scale.

**Table 3:** Prevalence estimates of chronic pain related to the initial ED pain, according to different definitions 3 months after the initial ED visit for acute pain (*N* = 1411).

Chronic pain operational definitions	%	95% CI
Patient self-reported suffering from chronic pain	23.3	21.1–25.5
Patient with pain intensity ≥ 1^∗^	45.3	42.7–47.9
Patient with pain intensity ≥ 4^∗^	22.5	20.3–24.7
Patient with pain intensity ≥ 4^∗^, every or most days	19.6	17.5–21.7
Patient with moderate or higher disability level (PDI)	27.6	25.3–29.9
Patient with pain intensity ≥ 4^∗^, every or most days with moderate or higher disability level (PDI)	16.9	14.9–18.9

Abbreviations: CI = confidence interval, ED = emergency department, PDI = Pain Disability Index.

^∗^Pain intensity on a 0–10 scale.

**Table 4:** Type of agreement between self-reported chronic pain related to the initial ED pain and different chronic pain operational definitions (*N* = 1411).

Chronic pain operational definition	Self-reported chronic pain	
	Yes	No
Patient with pain intensity ≥ 1^∗^		
Yes	22%	24%
No	2%	53%
Patient with pain intensity ≥ 4^∗^		
Yes	16%	7%
No	8%	70%
Patient with pain intensity ≥ 4^∗^, every or most days		
Yes	15%	5%
No	9%	72%
Patient with moderate or higher disability level (PDI)		
Yes	17%	10%
No	6%	66%
Patient with pain intensity ≥ 4^∗^, every or most days with moderate or higher disability level (PDI)		
Yes	13%	4%
No	10%	73%

Abbreviations: ED = emergency department, PDI = Pain Disability Index.

^∗^Pain intensity on a 0–10 scale.

**Table 5:** Opioid and NSAIDs/acetaminophen consumption 3 months after the initial ED visit, according to different chronic pain definitions (*N* = 1411).

Chronic pain operational definition	*N*	NSAIDs or acetaminophen (%)	Opioids (%)
Patient self-reported suffering from chronic pain	329	76	17
Patient with pain intensity ≥ 1^∗^	639	**64**	**11**
Patient with pain intensity ≥ 4^∗^	317	80	18
Patient with pain intensity ≥ 4^∗^, every or most days	277	82	19
Patient with moderate or higher disability level (PDI)	389	75	17
Patient with pain intensity ≥ 4^∗^, every or most days with moderate or higher disability level (PDI)	238	84	21

*Note:* Bold: statistically different from other definitions, *p* < 0.001.

Abbreviations: ED = emergency department, PDI = Pain Disability Index.

^∗^Pain intensity on a 0–10 scale.

## Data Availability

The data that support the findings of this study are available on request from the corresponding author. The data are not publicly available due to privacy or ethical restrictions.

## Supporting Information

These supporting tables provided baseline characteristics for participants with and without agreement between self-declared chronic pain and the other chronic pain operational definitions (Supporting Tables 1–4).
