# Supplementary material for: How Does Self-Declared Chronic Pain Compare to Other Definitions? A Prospective Multicenter Study
Source: Pain Res Manag. 2025 Jun 19;2025:5556400. doi: 10.1155/prm/5556400 (PMC12202084; doi:10.1155/prm/5556400)
Supplement: Supporting Information — Additional supporting information can be found online in the Supporting Information section. [file 5556400.f1.docx]

Supplement Table 1. Baseline characteristics for participants with and without agreement between self-reported chronic and pain intensity ≥ 1 definition (N=1,411).

| Baseline characteristics |  | Agreement (N=1,057) | Without (N=354) | p χ^2^ |
| --- | --- | --- | --- | --- |
| Mean (±SD) age |  | 51.6 (15.6) | 52.3 (15.8) | 0.46^a^ |
| Female (%) |  | 51.3 | 55.9 | 0.13 |
| Ethnicity group (%)  -White  -Other |  | 79.9  20.1 | 81.0  19.0 | 0.65 |
| Education (%)  -Primary/high school  -College/University |  | 34.5  65.5 | 35.1  64.9 | 0.82 |
| Type of pain conditions (%)  -Fracture  -Back pain  -Neck pain  -Other musculoskeletal  -Abdominal pain  -Other |  | 65.6  71.9  78.0  76.3  85.0  88.8 | 34.4  28.1  22.0  23.7  15.0  11.2 | 0.001 |

ED: Emergency department; ^a^: p from independent t-test.

Supplement Table 2. Baseline characteristics for participants with and without agreement between self-reported chronic pain and pain intensity ≥ 4 definition (N=1,411).

| Baseline characteristics |  | Agreement (N=1,210) | Without (N=201) | p χ^2^ |
| --- | --- | --- | --- | --- |
| Mean (±SD) age |  | 51.2 (15.7) | 55.3 (15.3) | 0.001^a^ |
| Female (%) |  | 51.5 | 58.2 | 0.08 |
| Ethnicity group (%)  -White  -Other |  | 80.3  19.7 | 79.5  20.5 | 0.79 |
| Education (%)  -Primary/high school  -College/University |  | 34.4  65.6 | 36.0  64.0 | 0.66 |
| Type of pain conditions (%)  -Fracture  -Back pain  -Neck pain  -Other musculoskeletal  -Abdominal pain  -Other |  | 81.2  80.1  90.0  85.6  94.0  96.6 | 18.8  19.9  10.0  14.4  6.0  3.4 | 0.001 |

ED: Emergency department; ^a^: p from independent t-test.

Supplement Table 3. Baseline characteristics for participants with and without agreement between self-reported chronic and pain intensity ≥ 4 on every or most days definition (N=1,411).

| Baseline characteristics |  | Agreement (N=1,215) | Without (N=196) | p χ^2^ |
| --- | --- | --- | --- | --- |
| Mean (±SD) age |  | 51.3 (15.7) | 54.7 (15.3) | 0.005^a^ |
| Female (%) |  | 51.6 | 57.7 | 0.12 |
| Ethnicity group (%)  -White  -Other |  | 80.6  19.4 | 77.4  22.6 | 0.30 |
| Education (%)  -Primary/high school  -College/University |  | 34.8  65.2 | 33.8  66.2 | 0.80 |
| Type of pain conditions (%)  -Fracture  -Back pain  -Neck pain  -Other musculoskeletal  -Abdominal pain  -Other |  | 81.9  81.5  90.0  86.3  93.2  95.1 | 18.1  18.5  10.0  13.8  6.8  4.9 | 0.001 |

ED: Emergency department; ^a^: p from independent t-test.

Supplement Table 4. Baseline characteristics for participants with and without agreement between self-reported chronic and moderate or higher disability level (PDI) definition (N=1,411).

| Baseline characteristics |  | Agreement (N=1,179) | Without (N=232) | p χ^2^ |
| --- | --- | --- | --- | --- |
| Mean (±SD) age |  | 51.5 (15.7) | 53.2 (15.8) | 0.15^a^ |
| Female (%) |  | 52.3 | 53.0 | 0.85 |
| Ethnicity group (%)  -White  -Other |  | 81.1  18.9 | 75.5  24.5 | 0.06 |
| Education (%)  -Primary/high school  -College/University |  | 34.4  65.6 | 35.8  64.2 | 0.69 |
| Type of pain conditions (%)  -Fracture  -Back pain  -Neck pain  -Other musculoskeletal  -Abdominal pain  -Other |  | 78.6  80.4  84.0  83.8  91.0  92.7 | 21.4  19.6  16.0  16.2  9.0  7.3 | 0.001 |

ED: Emergency department; PDI: Pain Disability Index; ^a^: p from independent t-test.
